# Are Health Information Systems Ready for the Digital Transformation in Portugal? Challenges and Future Perspectives

**DOI:** 10.3390/healthcare11050712

**Published:** 2023-02-28

**Authors:** Leonor Teixeira, Irene Cardoso, Jorge Oliveira e Sá, Filipe Madeira

**Affiliations:** 1 Department of Economics, Management, Industrial Engineering and Tourism (DEGEIT), Institute of Electronics and Informatics Engineering of Aveiro (IEETA)/Intelligent Systems Associate Laboratory (LASI), University of Aveiro, 3810-193 Aveiro, Portugal; 2 Associação Portuguesa de Sistemas de Informação (APSI), 4800-058 Guimarães, Portugal; 3 Department of Information Systems, Centro ALGORITMI, University of Minho, 4800-058 Guimarães, Portugal; 4 Department of Informatics and Quantitative Methods, Research Centre for Arts and Communication (CIAC)/Pole of Digital Literacy and Social Inclusion, Polytechnic Institute of Santarém, 2001-904 Santarem, Portugal

**Keywords:** digital transformation, health information systems, emerging technologies, Health 4.0, empirical study

## Abstract

Purpose: This study aimed to reflect on the challenges of Health Information Systems in Portugal at a time when technologies enable the creation of new approaches and models for care provision, as well as to identify scenarios that may characterize this practice in the future. Design/methodology/approach: A guiding research model was created based on an empirical study that was conducted using a qualitative method that integrated content analysis of strategic documents and semi-structured interviews with a sample of fourteen key actors in the health sector. Findings: Results pointed to the existence of emerging technologies that may promote the development of Health Information Systems oriented to “health and well-being” in a preventive model logic and reinforce the social and management implications. Originality/value: The originality of this work resided in the empirical study carried out, which allowed us to analyze how the various actors look at the present and the future of Health Information Systems. There is also a lack of studies addressing this subject. Research limitations/implications: The main limitations resulted from a low, although representative, number of interviews and the fact that the interviews took place before the pandemic, so the digital transformation that was promoted was not reflected. Managerial implications and social implications: The study highlighted the need for greater commitment from decision makers, managers, healthcare providers, and citizens toward achieving improved digital literacy and health. Decision makers and managers must also agree on strategies to accelerate existing strategic plans and avoid their implementation at different paces.

## 1. Introduction

Globalization, associated with many rapidly evolving factors, such as the COVID-19 pandemic, leads to an ever-increasing need to share health data outside of the physical space where they are generated [1,2]. Demographic changes, increased chronic diseases, rising health spending, and fairer healthcare access are global challenges [3], which, if associated with the increase in people’s average life expectancy and the growth in their literacy, show the greater importance of new Health Information Systems (HISs) that allow efficient communication between the Health Systems (HS) and their stakeholders. This has resulted in recent years in a new generation of emerging technologies that offer new opportunities for healthcare delivery and the practice of medicine while also ensuring greater efficiency of HIS and more responsive communication channels.

HIS includes mechanisms for capturing, processing, analyzing, and transmitting any needed information in health services whilst also having an important role in care planning, management, and even in research for public health [4]. With a growing need for decentralized and remote work, HIS also plays a key role since they support communication among geographically dispersed actors, promote solutions to value chain management, and support new business models. In addition, digital health offers a valuable opportunity to handle health issues, such as the pandemic situation, with near real-time responsiveness [5]. This trend is transversal to other areas of knowledge, with different types of applications, as demonstrated in the study of Epizitone et al. [6]. Information and Communication Technologies (ICT) applied to the health context have been the subject of several research works, with a greater incidence in the Digital Transformation (DT) of this sector [1,7], such as processes related to healthcare delivery models and medical practices [8]. Some studies point to digitalization as a future priority in the health and public sector, reinforcing the need to adopt intelligent technological applications [9,10,11,12] and connectivity mechanisms [13,14]. Cavallone and Palumbo [15], for example, stated that Industry 4.0 (I4.0), Artificial Intelligence (AI) and Digitalization are revolutionizing the design and the delivery of care.

Despite the progress observed in recent decades, the gap that emphasizes the need to create solutions with responses to extreme events is demonstrated by some authors [16,17]. In addition, Gehring and Eulenfeld [18] argued that there is still a pressing need to significantly improve the infrastructure and functionalities of the HIS for the benefit of users and also for research in areas such as biomedical sciences, health sciences, and also computer and information sciences.

Knowledge of the current HISs’ development state is a requirement when researching future directions. An analysis of current HISs [19] identifies five different groups of obstacles that limit these systems’ application and development—(i) technical problems, (ii) usage problems, (iii) quality problems, (iv) operational functionality, and issues related to (v) maintenance and support. In addition, the study of Khubone et al. [20] discussed a set of challenges that should not be ignored when adopting HIS, which are mainly related to the lack of technical consensus, poor leadership and limited human resource, staff resistance and lack of management, and non-engagement of the users. In the area of telemedicine, Tabaeeian et al. [21] compiled a set of barriers that should not be ignored when implementing such solutions, concluding that “the future of telemedicine depends on consistency in system usage and minimizing problems, increasing system compatibility with users and learning how to use”. In turn, the development of a HIS requires several connections between local systems, which can be small county or regional systems or national platforms, a connection between different sectors (such as public, private, and other), and can even need the articulation at an international level (e.g., between European countries, or USA and Canada).

There is a generally recognized importance of HISs by health policymakers. In Portugal, this is a reality reflected by the creation of the National Strategy for the Health Information Ecosystem 2020 (ENESIS-2020) [22]. This working framework elected the main goal to have more efficient information processes that would (i) increase the general sharing of information and knowledge between all actors to promote the literacy and general health of citizens; (ii) offer greater efficiency for healthcare providers; (iii) offer greater rationalization of resources with an impact on global efficiency and health management; and (iv) offer an alignment of HIS strategies with other European countries (i.e., standards and interoperability).

Considering the impact that these systems have on healthcare management and the medical practice, whilst also considering the current technological trends currently reported in the literature, this study aimed to understand the current state of HIS in Portugal and its main challenges, as well as foresee future trends about the use and impact of this type of systems that can benefit with emerging I4.0 technologies. The authors adopted a two-phase methodological approach. First, the authors carried out a literature review, covering topics related to the role of HIS with existing technologies, namely the ones that could enhance the development of these systems. A focus on digitalization technologies was pursued. Secondly, the results from the literature review were compared to the current Portuguese HIS situation. This was achieved by conducting an empirical study based on interviews with some representative HIS actors in Portugal while also considering some official documents, legislation, and strategies/policies currently in force in Portugal.

Theoretically, this work aimed to advance some likely future trends and scenarios for the Portuguese’s HIS based on the Industry 4.0 drivers. In practical terms, it was expected to provide some recommendations to practitioners and decision makers on the opportunities of DT and the expected main impacts on the Portuguese HIS. As an additional contribution, it was also intended to identify some aspects that would enable societal health empowerment through the adoption of emerging technologies.

This article is structured into six sections. The current section presents the gaps, the motivations for this study, and the main objectives to be achieved. In Section two, a literature review is conducted. The third section describes the methodology adopted in this study. Section four presents the main results, challenges, and future scenarios for HISs in Portugal obtained from the point of view of the study participants (i.e., the interviewees) and from documents, legislation, and strategies/policies analyzed. In Section five, the authors present their views on future challenges and scenarios for the Portuguese HISs. Finally, the last section is devoted to some final remarks.

## 2. Theoretical Framework

### 2.1. Evolution of HIS to Date

The practice of medicine has changed significantly in the last 50 years, with ICT making a strong contribution to this change. There have also been changes in the information paradigm itself, moving from institution-centered information to a more patient-centered approach [8].

Due to globalization and other circumstantial factors, such as the COVID-19 pandemic, there is an increasing need to share health data outside of the physical space where they are generated [1,2,20]. Furthermore, the change in the clinical information consumption patterns, where the citizen assumes an increasingly significant role, is also a reason that highlights the importance of HIS. The final use given to the data, which before was focused on responses to clinical practice, has gradually assumed greater importance also in health planning and clinical and epidemiological research.

**Figure 1:**
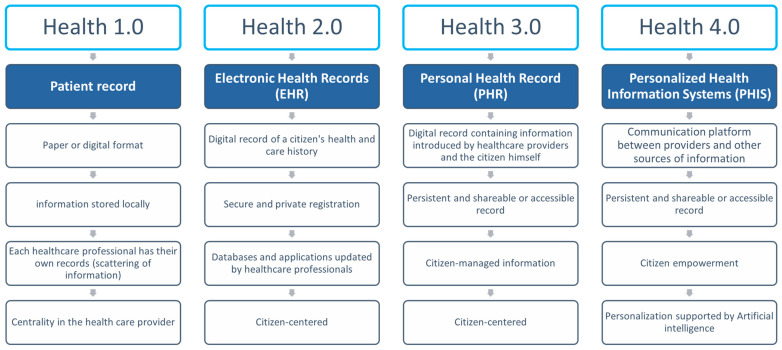
Evolution of HIS in health according to technological “eras”.

Over the past decades, HIS has had various stages, as briefly described in Figure 1, which presents HIS usage in health in four digital eras—from Health 1.0 to Health 4.0.

The first era, also called Health 1.0, began in the 1960s and was associated with the introduction of patients’ records. During this period, HIS was exclusively designed to store patients’ information locally, in paper or digital format. Access was limited and only available in each service, department, or institution.

Afterward, in the Health 2.0 era, which started in the late 1980s, HIS was further developed, so to allow the grouping of patients’ data in digital repositories with private access to authorized users and necessary services, materializing the Electronic Health Record (EHR). The information was then citizen-centered [23], increasing the role of patients/citizens in HISs as they started to have limited access to the information recorded by health professionals.

The Health 3.0 era, which began in the early 2000s, was characterized by the development of Personal Health Records (PHR). During this time, the main goal of HISs was to support the citizen’s life cycle, with data introduced by both healthcare providers and the citizen himself. This further advancement of HIS allowed patients to engage proactively and collaboratively in their care. Thus, data were co-created and maintained by both providers and patients [24], and society moved from institution-centered information to a more patient-centered approach [8]. PHR quickly became attractive, as it allowed the centralization of each citizen’s health data in digital platforms widely available, engaging both providers and receivers in healthcare deliverance.

Nevertheless, most countries and providers still felt that there was a need to have a connected decentralized health system. This was made possible by new communication platforms and emerging technologies, as well as the use of Artificial Intelligence (AI), developed during this past decade. Personalized Health Information Systems prevail, but with a vastly improved connectivity between healthcare actors, in what is called the Health 4.0 era.

### 2.2. The Fourth Industrial Revolution and the HIS

The creation of digital ecosystems through a set of tools that enable the connection between the digital and the physical worlds is paramount in Industry 4.0 (I4.0) technologies. This last concept, which is a product of the fourth Industrial Revolution (4IR), is intricately related to principles such as interoperability, decentralization, real-time responsiveness, data-based services, virtualization, and modularity [25]. I4.0 uses, for the technologies developed, a variety of concepts such as automation and data exchange through cloud computing, Big Data, Internet of Things (IoT), Robotics, 5G Technologies, Virtual and Augmented Reality, Additive Manufacturing, Cyber-Physical Systems, and AI, among others [25,26,27].

With the promise of empowering the creation of better healthcare services, 4IR enhances the personalization and individualization of the services provided, the optimization of resources associated with the practice of medicine, as well as the promotion of health based on preventive models [28,29]. Concepts include Health 4.0 [25,30,31] Healthcare 4.0 [32], Medicine 4.0 [33] or Care 4.0 [34], Hospital 4.0 [35,36], or more specific applications such as Surgery 4.0 [37], represent only some approaches/applications that make use of I4.0 emerging technologies to create new models of medical practice and health promotion. These concepts, in their different terminologies, represent just some extensions of the I4.0 principles applied to the medical/health area. 

Several authors considered that Health 4.0 could not be dissociated from the Digital Transformation (DT) concept, as the latter is used not only in the deliverance of care but also in the governance processes of all the value chains [38]. Health 4.0 makes possible the future virtualization of healthcare delivery and medical practice [31].

Connectivity and computing power, enhanced by emerging technologies, are crucial factors in the deliverance of need-oriented care, considering individualized approaches based on preventive and predictive models. The emerging technologies of I4.0, when applied to healthcare, can greatly enhance the productivity of the providers, as well as promote the creation of preventive care models since they allow for early detection of health-related anomalous situations [28]. It is then possible to avoid future health issues (and costs) for both citizens and society alike.

Some other applications include, for example, additive manufacturing or 3D printing, which allows faster and more personalized creation of health products, such as implants, tools, and specific devices, according to the different needs and requirements of each patient [39]; or robotics, which can be used in surgery and physiotherapy services, fostering improvements in performance, movement, and control. IoT allows connectivity with mobile and other devices, enabling the automatic collection of human data. Other examples include Big Data which, in addition to storing a large amount of data, allows, through Data Analytics, the identification of patterns and trends, enabling the decision-making on predicted problems of future health events. Finally, AI can help manage and analyze data, make decisions, and identify and forecast upcoming health trends or issues [40]. 

Recently, Large Language Models (LLMs) with billions of parameters have brought a big boost to the base models of deep learning, and several architectures have emerged, such as ChatGPT and BERT (which are among the best known). The research and interest around these deep learning technologies are huge and promising. ChatGTP, launched in late 2022, presents both great potential and challenges for the Medicine and Healthcare sectors.

AI-assisted technologies have for some time (and with different degrees of success) been employed in several aspects’ areas of healthcare. For example, in 2016, IBM launched Watson for Oncology [41], an AI clinical decision-support for cancer treatment, that achieved moderate success before being discontinued for failure to achieve the same clinical marks as real-time physicians [42]. Other examples come from radiology or pathology, where AI-assisted tools are being employed to identify tumors, differentiate between healthy and abnormal tissue samples, and provide clinicians with diagnostic suggestions, which lead to faster and more efficient results and prompt treatment [43,44,45].

So, if AI is not new to the healthcare sector, it has mostly been used in specific areas and as support for clinical decisions. ChatGTP is different! Firstly, it was not designed with a specific medical intention in mind. Secondly, it is widely available. Finally, it has shown a considerate level of clinical accuracy [46], e.g., achieving the passing threshold of the USMLE (the United States Medical Licensing Examination). These characteristics can lead to important innovations in the healthcare sector, namely in developed countries, where this sector is struggling to deliver good healthcare in an aging society:

- Triage of patients—LLMs, such as ChatGTP, can be used as primary points of contact between the patient and the healthcare system, triaging patients and decreasing the burden on the healthcare system. Moreover, these tools can also reduce clinical biases, providing a standard of consideration to every patient, independent of personal characteristics.
- Medical scribe functions—modern healthcare systems require the input by the physicians or their assistants of large amounts of data. LLMs can be used to help or reduce this workload, performing note-taking tasks and writing brief patient summaries and presentations. One such example is a recent Microsoft announcement that Teams would provide note-taking features for meetings [47].
- Diagnosis assistance—LLMs can become important tools to help clinicians to make an evidence-based differential diagnosis as unbiased tools that can be trained not only with large amounts of medical information but which can also be updated with the latest relevant data, including innovative academic studies or clinical trials.

LLMs can also drive innovation and competition in the healthcare sector. Medical Sciences are, by nature, an uneven market field, where the provider (physicians) have all the knowledge, and the user (patient) does not know what he/she is getting before the service is complete. The digital revolution of the past few decades has reduced this gap, both empowering patients with information and promoting health literacy in general. Just in Europe, it is estimated that half of the patients look for health information online [48]. LLMs, such as ChatGTP, can increase this trend, promoting more informed choices and leading to more demanding customers (i.e., patients). This has the chance to drive innovation and promote excellence across the medical field.

There are, nevertheless, some challenges. ChatGTP, for example, remains an imperfect tool, with the CEO of OpenAI, the company behind this tool, recently twittered that ChatGPT remains “incredibly limited”, and that “It’s a mistake to rely on it for anything important right now” [49]. Obstacles such as misinformation, artificial hallucination, data protection, or ethical questions remain relevant and should, if not limit, at least warrant some cautions in the use and dissemination of these tools. 

In conclusion, LLMs present significant opportunities for the healthcare sector, but a careful approach involving practitioners, patients, policymakers, and other relevant field professionals are needed before they become mainstream. With all these developments, the Health 4.0 concept is closer than humanity imagines. 

Thus, the main pillars of Health 4.0 (and its derivations) are framed within digital ecosystems and are focused on people, technologies, and co-design because it presupposes a change in hospital business models to an ever-increasing citizen-centered care provision. Technology insofar represents the drives that are at the basis of the Health 4.0 concept itself, and without which its implementation would not be possible. Finally, to co-design patients’ involvement is a requirement, not only in the HISs as active actors but also in the design and development of these systems, to allow their future participation [29].

## 3. Materials and Methods

Starting from the objective that supported this research (to understand the current state of HIS in Portugal and its main challenges, as well as foresee future trends), an empirical and exploratory study was carried out, supported by a qualitative methodological approach, whose research design is presented below. So, the research protocol starts with a comprehensive literature review to define the boundaries of the research subject and the research questions, as well as to build the interview guidelines. Next, an intentional sample was selected from a population of HIS users in the private and public Healthcare organizations and Governmental entities involved in the definition of the HIS strategy in Portugal. In the third phase, the fieldwork that consisted of the execution of the interviews, as well as the selection of strategic documents, was conducted. The fourth stage comprised the processing and analysis of data, and finally, the analysis of findings and the consequent production of conclusions. 

**Figure 2:**
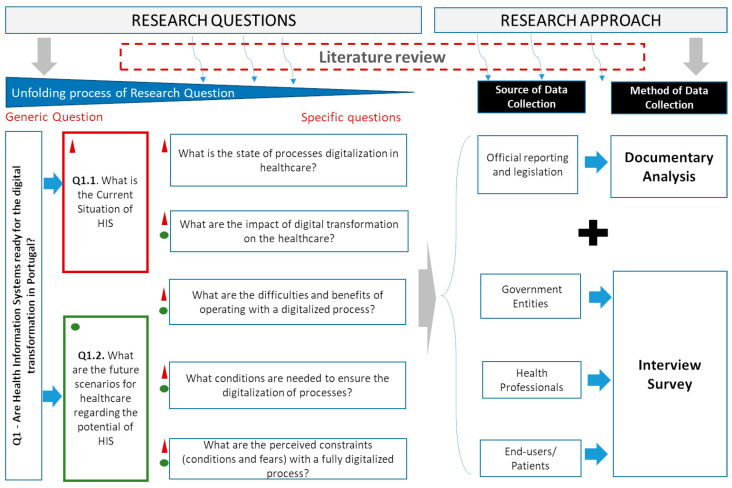
Research Design.

Figure 2 presents the research design followed in this study, where the main research question (Q1) was broken down into two sub-questions (Q1.1 and Q1.2), and these in other more specific questions. To collect data, different sources were used, which are broadly categorized as (i) analysis of strategic documents and legislation; and (ii) interviews. Strategic documents report a set of initiatives launched by government entities that can regulate and promote approaches for modernization in this sector. These documents included digital platforms, official documents, and legislation produced by the entities responsible for the definition of strategy and management of information on the health ecosystem at the national level, i.e., ENESIS-2020 [22] and ENESIS 20/22 [50]. The interviews were selected insofar, as they are considered one of the most proper methods to explore participants’ experiences and/or reconstruct past events.

### 3.1. Data Collection Methods and Procedures

As mentioned, the qualitative approach was adopted in the data collection, combining: (i) the analysis of strategic documents and legislation; and (ii) semi-structured interviews conducted with different entities involved both in the definition of the HIS strategy in Portugal and as users of these HISs. The interviews (audio-recorded) were applied between August and December 2019, following previously developed scripts, oriented and adapted to the interviewees’ profiles, and structured according to the specific goals mentioned in Figure 2. Each script included ten questions in addition to those that anonymously characterized the interviewee. Three types of interviewees were found, namely: (i) managers, which included health professionals with management or coordination positions; (ii) health professionals (physicians and nurses); and, also, (iii) users of HS. To conduct the interviews, the participant’s consent was obtained, and confidentiality and anonymity were guaranteed. All participants were interviewed in person by the researchers.

### 3.2. Data Analysis Methods

Due to the nature of the data obtained, qualitative analysis methods were used. Both the strategic reports and the transcribed interviews were subjected to a thematic-categorical content analysis, which represents a technique to capture the meaning of texts relating to a particular phenomenon under study [51]. For this purpose, the typical phases of content analysis were followed, which are based on: (i) the organization of the material and the definition of the procedures (pre-analysis); (ii) the identification of the categories that arise with the interpretation of the text (exploration); and finally, (iii) the treatment of the results, where we sought to interpret the data around the categories created. A manual coding procedure was used in this process.

### 3.3. The Sample Profile of Respondents

Given the exploratory nature of the study, intentional sampling was chosen. To minimize the limitations caused by the reduced sample size, particularly those related to the replicability and reliability of the study, we sought to diversify the demographic regions from where the participants originated. Table 1 presents the sample, composed of fourteen participants distributed as five health professionals (coded with the suffix P), six health professionals with management positions (suffix M), two users (suffix U), and one member of a governmental entity (suffix GE). Table 1 also presents other data that allow for a better characterization in terms of the region and organization to which they belong, the regime (public or private) in which they work, their profiles and positions, as well as their occupations and age groups. All interviewees mentioned being users of the HIS, although to different degrees.

## 4. Results

Based on the analysis of the interviews and some strategic documents [22,50,52,53], it was intended to answer the major research question Q1, see Figure 2. To achieve that goal, it is equally important to obtain answers to questions Q1.1 and Q1.2., i.e., to understand how the current state of the HIS in Portugal is as well as their trends in terms of future development.

### 4.1. Analysis and Reflection on the Current HIS

#### 4.1.1. Current HIS Situation: Document Analysis

From document analysis, which includes online platforms, and legal documents that approved strategies for HIS, for the last two three-year-olds [22,50], it was perceived that police decision-makers consider that IS Healthcare can act in any organization that involves healthcare (public and private hospitals, clinics, clinics, pharmacies, nursing services, and primary healthcare, among others).

According to the Resolution of the Council of Ministers of 26 July 2017 [53], the HIS seems to include “all local and central information subsystems, in the entities of the National Health Service (NHS) and third parties integrated with it, to make available to several users all useful information to health literacy and health self-management (citizens), providing healthcare (health professionals), system management (local and central managers), health research and cross-cutting needs for public administration” [53].

The main strategy for HIS, entitled Health Information Ecosystem Strategy 2020 [20,40] adopted by Council of Ministers Resolution No. 62/2016, replaced by ENESIS 20/22 [50], was approved on 7 January 2020 after public consultation. 

The ENESIS 2020 [22], as well as the following (ENESIS 20/22) [50], assume the objective of promoting the Digital Transformation of the health sector in Portugal and creating the conditions that allow the evolution of the Health Information Ecosystem (eSIS). They seek to respond to the priorities defined in terms of health policies, extending to the entire Health System and ensuring a common vision for the area of IS/IT. Analyzing the strategy still in place, ENESIS 20/22, the authors considered in general terms that it promotes a citizen-centered approach, ensuring simple and timely access to healthcare and improving his/her experience with the system [50]. The implementation of the strategy was structured in a set of six axes, namely: (i) access to healthcare throughout the life cycle of the citizen; (ii) training and empowerment of citizens; (iii) efficiency and sustainability of the health system; (iv) quality and safety of healthcare; (v) prevention, protection, and promotion of health; and (vi) training of professionals in organizations. Some of these axes (e.g., access to healthcare throughout the life cycle of the citizen; training and empowerment of citizens) seem in line with some literature which says that the healthcare sector begins to adopt a perspective less and is less based on hospital space and health professionals, and more focused on the citizen and their needs. Yet, in relation to the aspects such as efficiency and sustainability of the health system, quality and safety of healthcare, prevention, protection, and promotion of health, and training of professionals in organizations, they can find in other authors. 

Regarding the Health Information Ecosystem in Portugal and considering the results obtained from documental analysis (placing references to the websites and the legislation/strategy), the authors could verify that there was a comprehensive evolution of the HIS in Portugal, which followed the evolution of the HIS in general [8,24] and in several countries of Europe, which is reflected in its strategy. Adopting a holistic and citizen-centered view, the HIS in Portugal increasingly tries to respond to the information needs around the life cycle of the person, from birth to death, being visible in the definition of the Health Information Ecosystem (eSIS), “a set of technologies, people and processes that intervene in the life cycle of information related to all dimensions of the health of citizens (…) regardless of the place of care and/or organizational barriers [52] (p. 3736)”. It is precisely in this vision that the authors make the description of the HIS existing and/or under development in Portugal. 

In Portugal, many HISs can be found in various areas (Administrative and patient management, Clinical, Financial, Management and planning, Informative and IT, and Communication), according to SPMS [54]. However, the authors focused their analysis primarily on HIS connected with the life cycle of citizens. 

Thus, following a citizen-centric perspective, Table 2 presents the main HIS classified according to the different stages of people’s life cycle, i.e., birth; (ii) health and well-being; (iii) disease, which may be acute and/or chronic; (iv) aging; and (v) Death. Some of the HISs are transversal to the various stages of the life cycle, serving the citizen from birth to death, such as the ‘National Register of Users’ (RNU) and the ‘Electronic Health Record (RSE).

#### 4.1.2. Findings from Interviews

The results obtained from these data are presented according to the following themes that have already emerged from the literature review with the unfolding of the research question: (i) the state of digitalization of clinical information processes; (ii) the impact of DT on the HS; (iii) difficulties and benefits of operating with digitalized processes; (iv) conditions for dematerialization; and (v) reduction of info exclusion. 

##### The State of Processes’ Digitalization

The results pointed to two distinct groups of respondents’ thoughts. The first was satisfied when asked about the state-of-the-art of HIS in their organizations and claims to have achieved a significant digitalization through DT, as can be read in the following statements:

*The process of digitalization is almost consolidated in the public sector, (…), we have problems with very large hospitals with very old and poorly computerized systems, both at the regional and national levels that delay the process as a whole.*

*(E13-GE)*

*We are taking steps towards full integration in terms of system development (…). Portugal is far ahead, compared to many countries, in Europe.*

*(E11-M)*

*We are even implementing the paperless hospital project which is a project that has had good and referenced results, our hospital can get 70% of patients to leave the hospital without paper.*

*(E10-M)*

The second was more cautious in their statements, being more skeptical, saying that although there has been a significant advance in recent years, there is still a lack of integration and communication between systems, as interpreted by the following comments:

*(…) it has evolved into a strategic concept, but there is a long way to go, there is a lack of clinical information in the interaction between private and public institutions (…)*

*(E6-P)*

*(…) there is still a lot of lack of communication between hospitals and health centers. A lot of time is spent transcribing the analyses (…) it is necessary to continuously improve the software (…)*

*(E3-M)*

##### Impact of Digital Transformation on the Healthcare

In general, respondents saw digital health as a way to promote health. One interviewee referred that DT supports another way of doing medicine and promoting citizens’ health with an impact on the creation of new business models in clinical practice.

*Digital health is another type of (…) health service, and another way of doing health” (…) to telehealth, but is connected for example with preventive medicine, with precision drugs.*

*(E13-GE)*

However, and in general, despite the potential benefits that the interviewees see in the eventual digitalization of processes, they were also unanimous about the challenges that DT brings. Thus, some benefits and challenges emerged, as can be seen in the following comments:

*These platforms can complement and help diagnosis (…)*

*(E1-U)*

*The potential to do good, to change health (…) the transformation of health, from one-on-one health practice to a population health practice*

*(E11-M)*

*There are already positive impacts, but the centrality of the patient in the system does not exist*

*(E6-P)*

##### Difficulties and Benefits of Operating with Digitalized Processes

As regards difficulties in the operationalization and use of systems, some respondents with management responsibilities reported difficulties in the human resources area related to resistance to change. Additionally, difficulties associated with the functioning of the HIS, namely: slowness, redundancy, lack of response to clinical practice, and lack of interoperability (communication and integration) between public and private institutions, were also pointed out by some respondents:

*(…) it has more to do with people than with technology (resistance to change, stability of teams, continuous training)*

*(E11-M)*

*(…) lack of integration of systems between institutions*

*(E2-P)*

*(…) there is no standardization of the systems themselves, they are always different systems*

*(E4-P)*

Regarding the expected benefits of digitalization, respondents (E3-M) (E4-P) (E5-M) (E10-M) perceived some benefits in the digitalization process, such as (i) greater agility in information flows, with access to information in almost real-time; (ii) more security, in terms of access to data only by authorized users; (iii) less propensity to human error; (iv) greater capacity to share information between services and organizations providing of healthcare; (v) easier of access to health services without geographical restrictions; (vi) reducing paper circulation and consumption, and (viii) cost savings often associated with the repetition of exams to better understand everything that surrounds the activity of healthcare. 

##### Conditions for Digital Transformation

The results pointed to the need to work on the aspects related to legislation, namely with governmental entities of each country and even outside the country. For example, in the sharing and use of health data from wearable devices, the greatest trouble is with the issues of use and legitimacy. With the technologies currently available, citizens can generate data to complement their health records through smart devices. To support the use of these technologies for health, legislation and some joint work with health regulators is needed. The results of the interviews also suggested the need for standards to achieve the interoperability of systems at the international level.

*(…) it is necessary to create legitimacy to define the use of digital technology [wearables] because it is different if I use it to record my health data, or if the data generated by these technologies can be used to make diagnoses or suggest therapy. This is too important to be seen at an international level to define interoperability standards and rules*

*(E13-EG)*

The ENESIS-2020 [22] and ENESIS-20/22 [50] assume the objective of promoting the DT of the health sector in Portugal and creating the conditions that allow responding to the priorities defined in terms of health policies, extending to the entire HS and also privileges a citizen-centered approach, ensuring simple and timely access to healthcare and improving his experience with the system. 

##### Conditions Required for the Reduction of Info Exclusion 

When it comes to ensuring the digitalization of processes, the question arises about citizens’ digital literacy, which may represent an enabling factor or an obstacle in the functioning of processes in that ecosystem. The reduction of info exclusion was referred to by 12 (86%) of interviewees as an important challenge to overcome.

On this subject, the interviewees reported that the reduction of illiteracy depends on several factors, namely: (i) the opening of the user to this type of knowledge and innovation; (ii) the development of accessible systems; and, also, (iii) the monitoring and training of older people and/or those with greater difficulty to ensure health literacy and digital literacy. The responsibility for this training and monitoring would be on the government, health organizations, educational institutions, and community entities, such as the town halls and parish councils. Supporting these findings, we have the comments presented below:

*(…) the current population is very ageing (…) it does not easily adapt to IT. The state should ensure minimal training, monitoring, and simplify the development of these technologies (…)*

*(E1-U)*

*(…) we have pioneering projects such as Citizen HOSP that, through our social workers, support users to take advantage of the use of IT in access to health services (…)*

*(E10-M)*

The implementation of ENESIS-2020 [22] and ENESIS20/22 [50], developed in recent years, is still in the consolidation phase, presenting, however, a significant advance in terms of the number of new digital services, namely digital platforms such as the Health Web Portal and other applications that can be accessed from the Citizen Web Portal.

These systems, in turn, tend to respond to certain stages of a citizen’s life, ranging from the simple registration of citizen follow-up to systems that accommodate the entire clinical history when in situations of illness (severe or chronic). This concept appears implicitly in ENESIS-2020 [22], defining it as a set of technologies, people, and processes that intervene in the life cycle of information related to all dimensions of citizen health and related, regardless of the place of care and/or organizational barriers.

### 4.2. Prospects for HIS in Portugal and Scenarios

Looking at future scenarios and based on the analysis of the results, three categories were shown that allowed to outline some scenarios for the future of the HIS in Portugal (i) Medicine Practices; (ii) Technologies; and (iii) Fears and Challenges.

#### 4.2.1. Medicine Practices

The way the interviewees saw the evolution of the practice of medicine associated with technological evolution was dual. If, on the one hand, they understand that technologies combined with the greater use of AI will make medicine richer, more dematerialized, advanced, and effective (namely by speeding and accuracy in diagnosis, self-learning, and knowledge because it is based on predictability and allows personalized treatments), with an impact on the increase in quality average life expectancy, on the other hand, they consider that these benefits cannot imply the loss of the doctor–patient relationship nor can the data overlap to the psychological reality of the patient in the interpretation of his disease.

*(…) Reduction of doctor-patient contact because computer solutions will compare certain standards by AI and allow diagnostics, without the patient presence. (…) a great combination of general medical knowledge with computer knowledge*

*(E1-U)*

*The practice of medicine in the future will be more dematerialized, remote[telemedicine], a preventive and precision medicine (…) the citizen will be more involved in his/her health/disease and the decisions about it, he/she will now his/her test results, and he will already bring the data stored in digital media.*

*(E13-GE)*

The last comment highlighted the core of current health strategies and policies, which focus on the citizen/patient, and the centrality of the patient with his/her information would enhance preventive models in health and accuracy in personalized diagnostics and treatment. 

However, it should be noted that the results also identified a gap between the progression of technology and the way healthcare is organized, the latter being associated with strategy, management, and legislation:

*(…) technology is evolving and the way we organize ourselves to supply healthcare is not advancing at the same pace.*

*(E13-GE)*

#### 4.2.2. Technologies

On this subject, some health professionals were skeptical about the adoption of technologies in medical practices, believing in a worsening of the social part, with a negative impact on the patient–doctor relationship, contrary to other professionals who had an optimistic view of the future of HIS.

*(…) there has been a decrease in doctor-patient confidence and (…), this system, although useful, can aggravate even more this situation*

*(E9-P)*

*(…) we need robust systems to treat this information, such as Business Intelligence or Data Mining, which are being implemented in our hospital (…) technologies allow us to innovate health, better manage resources, know patients (…)*

*(E10-M)*

*In ten years, I think it will be possible to computerize almost total medical information*

*(E9-P)*

Some interviewees, such as (E13-GE) and (E1-U), went further in what they think is the future of HIS, referring to the decrease in interfaces between technology and users, the existence of speech recognition software to support health professionals filling EHRs, the increasing existence of intra-devices, and the storage of data by patients themselves for reasons of cybersecurity, privacy, and data sharing.

#### 4.2.3. Fears and Challenges

Faced with the idea of a fully digitalized reality, most respondents mentioned concerns about the confidentiality of information (who accesses the data and with what intention), as well as other types of threats (e.g., cyberattacks). The dehumanization in the provision of care also arises as an apprehension that stems from the reduction of physical contact between doctors and patients (for example, health professionals can make decisions based on a set of data without the need for the physical presence of the patient), thus losing the emotional aspects characteristic of human interaction, typical of traditional medicine. The comments presented below reflect these fears:

*(…) lose the patients’ data (…)*

*(E2-P)*

*(…) who has access to this information and what are you going to do with it?*

*(E11-M)*

*Some of the pointed-out fears and negative opinions about ICT evolution can be seen as challenges by HIS developers. The following comments can be illustrative:*

*(…) doctors must rediscover themselves, as coachers, people who guide reading*

*(E13-GE)*

*(…) the data still cannot sustain the psychological reality of the patient in the interpretation of his disease (…) we must not forget that we have biological complexity and that the Human being is not purely data. We do not treat data, we treat people*

*(E6-P)*

#### 4.2.4. Possible Scenarios

Based on the previous categories and following the three dimensions identified and described above, some relevant concepts were found, which allowed the design of future scenarios for the HIS.

(i) Medical Practices—the following concepts were found:
  - Precision Medicine/Individualized—a medicine whose treatment is specific to a particular patient.
  - Preventive Medicine—in which the focus is to keep healthy instead of curing the disease.
  - Point-of-Care (Telemedicine)—allowing citizens to be physically distant from medical centers to have access to expert diagnoses.
  - Assisted Medical Practices—in which machines (robots) with AI embedded start to help or even replace health professionals in various medical acts.
(ii) Technologies—the concepts found are:
  - Interoperability—integration of HIS with intra- and inter-organizational information exchange between public and private and national and international entities.
  - Digital Health Transformation—health processes are aided by technologies.
  - Technology to Assist Medical Practices—such as robots and AI, among others, helping health professionals.
  - Use of electronic devices (wearables), robotics, and intra-devices—used by citizens to monitor their health, with the possibility of collecting data and sending these data to health entities/health professionals.
(iii) Challenges and Risks—the concepts are:
  - Resistance to change (people, users, and health professionals)
  - Information exclusion (Training/Monitoring)
  - Information (privacy, quality, and security—access and loss)

Given these perspectives, three HIS scenarios were developed for the future, i.e.,: (i) realistic; (ii) pessimistic; and (iii) optimistic scenarios.

Table 3 describes the scenarios for Medical Practices.

Table 4 shows the concepts related to Technologies.

Table 5 outlines the scenarios related to Challenges and Risks.

## 5. Discussion and Reflection

The health sector has, from early on, incorporated the benefits of the use of information technologies, with the first era of HIS that supported caregivers in their practice arising in the 1960s. The evolution has been remarkable, and the EHR, initially held by healthcare providers, is now run by citizens, true owners, and interested parties of their health data. However, this change requires an effort from all agents in terms of the design of “future” HIS, where aspects such as interoperability, standardization, privacy, security, and actions to address info exclusion appear as striking challenges. These factors and challenges are shared by the respondents, as shown in Table 6. However, a greater or lesser sharing should not be understood as a greater or lesser importance of each of the challenges but rather demonstrates the degree of awareness of these factors and challenges by the group of respondents. 

This change has been driven by social factors, such as increased life expectancy and mobility, but also by a set of ambitious responses and strategies created by healthcare decision-makers and managers. The reinforcement and commitment to HIS are expected to provide an adequate response to the health of citizens and guarantee the overall sustainability of the system. Thus, the technological advances brought by the 4IR have had a significant impact and acceptance in this sector, and the pandemic has further accelerated its adoption. The Health 4.0 concept incorporates innovative technologies which promote substantial improvements in health services and facilitates a more citizen-focused model concerning health.

The implementation of HIS in Portugal takes place at different paces, depending on the areas of activity, the type of sectors involved, and the legal regime of the organization, among other factors. It should also be noted that existing strategic documents specifically cover the public health sector and therefore do not consider private health entities; although the proposed ENESIS-20/22 strategy refers to a wider health ecosystem, in practice, the approaches presented are very much public-sector-focused. 

In line with Ciasullo et al. [29], to make the HS sustainable, it is necessary for citizens to actively take part in their health process, and to do so, they need to have adequate means and knowledge, as well as the ability to interpret their health data, which requires an investment in their digital and health literacy. Likewise, health organizations, public or private, together with industry regulators and those responsible for health strategy, need to work together to enable (digital) communication and integrated sharing of health data, particularly in the context of healthcare.

In addition, emerging I4.0 technologies have enhanced the creation of better conditions for data collection and information sharing, as reported by some studies [25,26]. Nowadays, there are already several types of equipment that can be used or applications that can be installed on personal mobile devices, which allow monitoring the parameters of health and quality of life [28]. It is, therefore, a pressing thing to evaluate and classify these types of devices and applications in terms of quality and reliability and to legitimize them for this purpose so that citizens and health professionals can see their usefulness, have confidence in their use, and can use them by increasing preventive and predictive health models.

Given the current technological context, the citizen, in addition to standing as a fundamental actor in data creation, can also play a relevant role as a consumer of information. As such, it is also important to provide citizens with access to health-related information, for example, through the EHR, thereby enhancing better decisions about their health while promoting a preventive health model, concerns already seen in other studies that seek citizen centricity [31,35].

Another important aspect that was highlighted in this study is the need to raise awareness and training of the citizen so that they are the main promoters of their health which confirms the results presented by Rahi et al. [55]. Thus, it is necessary to ensure that the citizen has conditions for this, such as (i) empowering the citizen to use the systems; (ii) raising citizens’ awareness to manage their health data, as well as sharing them with the professionals responsible for monitoring them, in a digital, holistic, and integrated ecosystem; and (iii) empowering citizens on the correct use of digital health solutions. 

The existence of health data, part of them collected by the citizens themselves, as well as the later exchange of this data between patients and health professionals, would certainly allow an increase in the value of the services, enhancing benefits for both parties. It is essential to ensure reliable and quality data sources, as well as their protection, privacy, and security.

In a more social and human aspect, and in line with what has been proposed in several other studies [1], it is important to highlight the importance of health professionals with adequate skills to implement DT in the health sector and, consequently, develop new processes in terms of healthcare and/or restructure existing ones, as well as the training of citizen so that they are the main responsible and promoters of their health. 

To conclude, several new trends, described around three scenarios, which may emerge in the future with the adoption of emerging I 4.0 technologies, should be noted, although these require a new strategic approach, with appropriate action plans to promote accessibility for all citizens and professionals.

### 5.1. Theoretical Implications

This study reinforced the literature’s long-held view about the importance of future health promotion strategies through I4.0 emerging technologies. The findings of this study confirmed that the digitalization of processes in healthcare can bring benefits to stakeholders while also bringing some challenges that should be properly addressed beforehand to maximize positive results. Furthermore, these findings could be used by researchers in Business Management and Information Systems areas to advance novel solutions to e-health-related sectors.

### 5.2. Managerial and Societal Implications

This study has several implications that are useful not only for health providers and receivers but also for society in general. Our findings showed that there is a need for greater commitment from managers and decision makers to invest in solutions that allow a more equal DT approach between different health institutions/sectors. In addition, decision makers should promote processes that not only support interoperability, respecting data privacy and security but also increase the digital literacy of all healthcare providers and the health literacy of system users.

## 6. Conclusions and Recommendations

This study assessed the current state of HIS in Portugal, verifying that there is a strategic alignment with our European partner countries in terms of legislation and definitions of health policies. Although there has been continuous redesign and the emergence of several applications/solutions for different processes, Portuguese health systems remain incomplete, with gaps to be filled in legislation and the adoption of innovative health-care processes by organizations. Moreover, struggles with integration and interoperability between solutions from the public and other sectors (private and social), or even from the same institution, not only lead to substantial costs in terms of redundancy and consistency of information but also reduce the interaction with users (healthcare providers, managers, and citizens). There were some other issues found, namely the need to improve digital literacy in all actors involved, as well as the urgency to increase citizens’ health literacy, both tasks requiring significant educational effort. Additionally, there remains some resistance to change. Nevertheless, the benefits expected (some already verified) by the different parties involved, such as the dematerialization, digitalization, and incorporation of emerging technologies, showed that there is an effective process of health DT in Portugal.

When it comes to the future of HIS, three possibilities which include a pessimistic, optimistic as well as a more realistic scenario, were outlined based on the Portuguese case. These fell into three main categories: (i) Medical Practices; (ii) Technologies; and (iii) Fears and Challenges.

### Limitations and Future Work

Firstly, a limited number of interviews were included. Even though an attempt was made to have individuals from different professional backgrounds and geographical areas, the results stood for a fraction of each sector’s professionals, and carefulness was warranted when generalizing the results.

Moreover, important, the interviews were performed before the COVID-19 pandemic, so the consequent increase in health’s DT was not considered, meaning that the results might not mirror the post-pandemic reality. Another limitation assumed by the authors is the fact that the results achieved and reported here came from qualitative research only, and there was no data to quantify the results.

As such, the authors would like, in the future, to re-evaluate the Portuguese HISs, checking the impact of the pandemic on the users’ health literacy and HISs’ development, using a more comprehensive method of data collection, with emphasis on quantitative approaches to data collection and analysis. In addition, it would be interesting to understand if the coronavirus outbreak forced a greater articulation of HISs between the public and private sectors. Finally, it should be noted that considering the utmost importance of issues related to data protection, privacy, and security of health data, it would be interesting to extend this study to evaluate questions related to these themes.

## Figures and Tables

**Table 1:** Characterization of the sample profile of respondents.

Interv.	Region	Organization	Regime	Profile (Position)	Profession	Age
E1-U	Azores	Praia and Vitoria Health Centre	Public	User	Computer Technician	--
E2-P	Azores	Praia and Vitoria Health Centre	Public	Professional	Doctor	41–51
E3-M	Azores	Praia and Vitória Health Centre	Public	Professional (Clinical Director)	Doctor	<40
E4-P	Azores	Praia and Vitória Health Centre	Public	Professional	Physician	<40
E5-M	Algarve	Family Health Unit Sol Nascente	Public	Professional (Hospital Coordinator)	Doctor	>51
E6-P	Algarve	Lusíadas Hospital	Private	Professional	Doctor	>51
E7-M	Aveiro	Finess Medical Clinic	Private	Professional (Clinical Director)	Doctor	>51
E8-P	Aveiro	Finess Medical Clinic	Private	Professional	Nurse	<40
E9-P	Aveiro	Tâmega e Sousa Hospital Centre	Public	Professional	Doctor	<40
E10-M	Aveiro	Ovar Hospital	Public	Professional (Chairman of the Board of Directors)	Manager	41–51
E11-M	Lisbon	Cascais Hospital	PPP	Professional (Chief Medical Information Officer)	Doctor	41–51
E12-M	Lisbon	Cascais Hospital	PPP	Professional (Chief Nursing Information Officer)	Nurse	41–51
E13-GE	Lisbon	Shared Services of the Ministry of Health	Public	Government Entity (President)	Manager/Doctor	41–51
E14-U	Faro	Retired	Public	User	Nurse	>51

**Table 2:** Health Information Ecosystem in Portugal.

Categories of Ecosystem of His (ESIS)	Designation	Description
Transversal of all NHS (National health service)	RNU; SER; SNS24; SINAVE	These HIS have the role of centralizing and distribution of information for NHS users
Life Cycle—Birth	Birth News; to Birth Citizen, Health Child’s Bulletin and Youth’s Health Bulletin and, eBulletin of Vaccines.	To receive a new citizen in society and, to monitor him/she in terms of surveillance and/or monitoring of public health
Life Cycle—Health and Wellness	Daily of My Health, SISO, SIIMA; SClínic CSP, RENTEV and others	This cycle comprises the systems that accompany citizens in a perspective of prevention and promotion of health
Life Cycle—Acute or Chronic Disease	ICC, Sclinic Hospital, SClinic CSP; RCU2, SINAVE, PEM, SI VIDA; RNCCI; and, CNTS	These HIS serve to accompany the user in his/her disease process, allowing the recording, diagnosis, and treatments, in all clinical episodes
Life Cycle—Aging	RECM; RNCCI.	HIS is intended to support clinical practice in the adoption and maintenance of healthy life models by the elderly
Life Cycle—Death	SICO	The main objective is dematerializing the process of certification of deaths and better articulation between the entities involved in the process.

**Table 3:** Scenarios for Medical Practices.

Medical Practices	Pessimist	Realist	Optimistic
Precision Medicine/Individualized	The costs (financial and adaptation) are enormous, and for this reason, it will not be the usual practice.	It will be used to solve serious and critical diseases, where the cost/benefit justifies it.	Medicine will be fully focused on the citizen, with better accuracy in the personalized diagnoses and treatments.
Preventive medicine	Those responsible for the health area still have difficulties adapting to a reality focused on prevention.	Health officials will try to make health digital, with a focus on health rather than a disease, optimizing the entire HS.	The practice of medicine is focused on prevention and health promotion.
Point-of-Care (Telemedicine)	It is already a current practice when distance obliges. One should bet on its development.	Telemedicine will be used regularly, regardless of distance, and more focused on solving the problems of the citizen.	Telemedicine will be used frequently, facilitating the sharing of information between professionals for cases of complex diagnosis, and the citizen will have privileged consultations with healthcare professionals through Telemedicine and Telehealth.
Assisted Medical Practices	Healthcare professionals will have digital assistants who will help make diagnoses, but the presence of the health professional will be required.	Healthcare professionals, in some diagnoses, will be replaced by machines. The use of machines (robots) to help some medical practices (e.g., surgeries) will be more common.	The diagnoses will be made by machines, and these machines (robots) will replace healthcare professionals in clinical practices, such as surgeries.

**Table 4:** Scenarios for Technologies.

Technologies	Pessimist	Realist	Optimistic
Interoperability (integration)	Health organizations (public and private), due to the existence of legacy systems or heterogeneous HISs, do not allow interoperability of the systems. Thus, the sharing of a citizen’s health data between several entities will be a distant reality.	Health organizations (public and private) collaborate in defining a set of shared services that allows the integration and access of a citizen’s EHRs.	HIS providers adopt international, European, and national recommendations, enabling interoperability between existing HISs and facilitating the sharing of EHR between different health organizations, respecting existing (legislation) standards.
Digital Health Transformation	It will occur when health organizations/entities (public and private) can change/innovate their processes, improve their leadership, and reduce resistance.	There are health organizations/entities (public and private) that innovate their processes, achieving significant efficiency gains. These cases will be examples to follow by other entities.	Health organizations/entities (public and private) present advanced dematerialization with significant gains in process performance. Success stories are shared and replicated.
Technology to Assist Medical Practices	Gradually technology that incorporates intelligence will be applied in the HIS; there is a need to create legitimacy for this to happen. Health professionals will resist but will eventually adopt the technologies.	The technology is currently able to assist professionals in medical practices. However, there are still obstacles to overcome: legitimation (legislation) and acceptance by all involved (health professionals and citizens) of the existed possibilities and limitations.	Health organizations and professionals perceive the positive side of incorporating intelligent technology and force legitimation (legislation) to occur. Intelligent technology, being incorporated into all medical processes and practices, leads to a huge efficiency gain and cost reduction.
Use of wearables	There are more and more devices able to collect data on citizens’ health. However, these data will not be used without regulation to process it. On the other hand, the existent healthcare services do not have the capacity to treat such data.	The collection of device-generated data is already a reality, and it does not raise technical issues; it is a matter of work, standards, and interoperability. The legitimacy of these systems and devices will occur, and healthcare models will adapt to this reality.	In the short term, legislation will be created to enable the collection and use of health data from electronic devices. Clinical practices will already use these data to promote models of healthy living for citizens.

**Table 5:** Scenarios for Challenges and Risks.

Challenges and Risks	Pessimist	Realist	Optimistic
Changing the existing culture (resistance to change among users and health professionals)	It will only occur when all stakeholders can understand the benefits to be obtained and realize that they will have to change/innovate their processes, and this will be a time-consuming process.	Health and care processes need to be innovated. There is little research and literature in this area. It is necessary to study the way care is organized and identify advantages and benefits causing changes in culture. Technology is a means and not the solution.	Healthcare delivery models will be studied and changed by accommodating emerging technologies with a positive and high impact on citizens’ health.
Info Exclusion (Training)	There is a need to simplify and disseminate the HIS, mainly those that are in place and those that will appear in the future, and the advantage of their use (to health professionals and citizens). It will be necessary to reduce digital illiteracy, mainly among older people.	Copying good practices successfully implemented by some organizations (training, monitoring, and involving all stakeholders), showing the advantages/benefits of using it.	All entities realize the advantages/benefits of using technological solutions and increasingly seek technological solutions to solve their problems.
Information (privacy, quality, security)	Legislation is needed to regulate the collection, access, treatment, and security of health information. This will be one of the biggest challenges of the next years.	The question of legitimacy (legislation) will be resolved quickly (by national or European directives). The next step will be to ensure the quality and security of this information so that all stakeholders maintain confidence in it.	The collection, access, and sharing of health data will already be sufficiently regulated to maintain high standards of data security and privacy. All stakeholders (within their legitimacy) can add and share health information with confidence with other entities.

**Table 6:** Important factors in HIS.

Key Factors to Address	Number of Interviewers Who Referenced It
Interoperability	4 (29%)
Standardization	3 (21%)
Privacy	6 (43%)
Security	7 (50%)
Actions to address info exclusion	12 (86%)

## Data Availability

Data sharing not applicable.
